# Phase 1 dose-escalation study evaluating the safety, pharmacokinetics, and clinical activity of OBI-3424 in patients with advanced or metastatic solid tumors

**DOI:** 10.1038/s41416-023-02280-4

**Published:** 2023-05-12

**Authors:** Apostolia Maria Tsimberidou, Claire F. Verschraegen, Robert Wesolowski, Chi-Sheng Shia, Pei Hsu, Tillman E. Pearce

**Affiliations:** 1 Department of Investigational Cancer Therapeutics, The University of Texas MD Anderson Cancer Center, Houston, TX USA; 2 Division of Medical Oncology, The Ohio State University Comprehensive Cancer Center, Columbus, OH USA; 3 OBI Pharma Inc., Taipei City, Taiwan; 4 OBI Pharma USA, Inc., San Diego, CA USA

**Keywords:** Targeted therapies, Targeted therapies

## Abstract

**Background:**

Report of a Phase 1 dose-escalation study of OBI-3424 monotherapy in patients with advanced solid tumors (NCT03592264).

**Methods:**

A classic 3 + 3 design was used to determine the maximum tolerated dose and recommended Phase 2 dose (RP2D) of OBI-3424 administered intravenously, as a single agent, at doses of 1, 2, 4, 6, 8, or 12 mg/m^2^ (days 1 and 8 of a 21-day cycle, Schedule A) or 8, 10, 12, or 14 mg/m^2^ (day 1 of a 21-day cycle, Schedule B).

**Results:**

Dose-limiting hematologic toxicities at 12 mg/m^2^ in Schedule A led to dose and schedule modifications (Schedule B). In Schedule B, maximum tolerated dose was not reached at the maximum dose tested (14 mg/m^2^). Grade ≥3 anemia was noted in 3/6 patients treated at 14 mg/m^2^; the RP2D was 12 mg/m^2^ (Schedule B). Grade ≥3 treatment-emergent adverse events were experienced by 19/39 (49%) and included anemia (41%) and thrombocytopenia (26%); three patients experienced serious treatment-emergent adverse events (grade ≥3 anemia and thrombocytopenia). One patient had a partial response and 21/33 (64%) had stable disease.

**Conclusions:**

The RP2D is 12 mg/m^2^ once every 3 weeks. OBI-3424 was well tolerated; dose-dependent, noncumulative thrombocytopenia and anemia were dose-limiting.

## Introduction

Aldo-keto reductases (AKRs) are a superfamily of NAD(P)(H)-dependent oxidoreductases that primarily catalyze the reduction of aldehydes and ketones to their corresponding alcohols [1–3]. AKR family 1 member C3 (AKR1C3), also known as 17β-hydroxysteroid dehydrogenase and prostaglandin F synthase, is involved in the synthesis of steroid hormones, catalyzing the reduction of androstenedione to testosterone and estrone to estradiol, as well as the synthesis of prostaglandins. AKR1C3 catalyzes the reduction of prostaglandin (PG)H2 to PGF2 and PGD2 to 9,11-PGF2. PGF2 and 9,11-PGF2 indirectly activate mitogen-activated protein kinase (MAPK) and inhibit peroxisome proliferator-activated receptor γ (PPARγ), resulting in cell proliferation [1–3].

AKR1C3 is expressed in various normal human tissues, including sex hormone-dependent (e.g., testis, breast, endometrium, and prostate) and sex hormone-independent (kidney and urothelium) tissues [2]. AKR1C3 is overexpressed in various solid tumors and hematologic malignancies, with strikingly elevated intensity in certain tumors (e.g., breast, prostate, endometrium, gastrointestinal tract, pancreas, liver, and kidney) relative to normal tissues [2].

In a population-level survey of 2490 patients across 19 cancer types, in which tumor tissue microarrays were used to assess AKR1C3 overexpression, the highest frequency was noted in hepatocellular carcinoma, followed by bladder, breast, prostate, renal, gastric, cervical, colon, and non-small cell lung (NSCLC) cancers [4]. Lung cancer showed marked heterogeneity, with no AKR1C3 overexpression in small cell lung carcinoma and high expression in lung tumor metastases [5]. In patients with NSCLC, AKR1C3 tumor overexpression has been associated with poor overall survival [5] and resistance to both radiotherapy and chemotherapy [6]. AKR1C3 overexpression in radiation-resistant NSCLC is associated with the function of AKR1C3 as a reactive oxygen species scavenger; notably, the efficacy of radiotherapy depends on the presence of reactive oxygen species [1, 6, 7]. AKR1C3 plays a role in carbonyl metabolism of a broad range of endogenous and exogenous substrates [2] and has the capability of reducing carbonyl-containing anticancer drugs, such as doxorubicin, into their related alcohols, thereby destroying their anticancer effect [1, 6, 8].

**Fig. 1:**
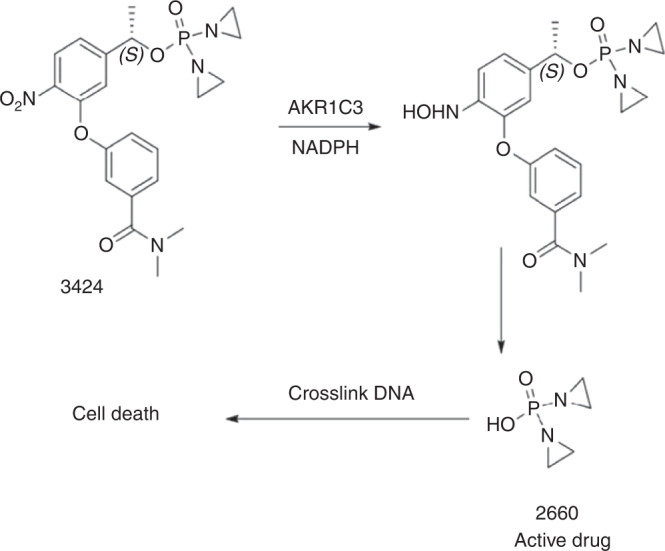
Reduction of OBI-3424 by AKR1C3 in the presence of NADPH to produce the cytotoxic agent OBI-2660. In the presence of NADPH, reduction of OBI-3424 is mediated by AKR1C3 to release the cytotoxic moiety OBI-2660, which is an aziridine bis-alkylating agent, leading to crosslinking of DNA at the N7 (or O6) position of guanine and subsequent cell death. AKR1C3 aldo-keto reductase family 1 member C3.

OBI-3424 is a highly potent DNA-alkylating prodrug that is selectively activated by AKR1C3 (Fig. 1). In the presence of NADPH, OBI-3424 is reduced by AKR1C3 to an intermediate that spontaneously hydrolyzes to the cytotoxic moiety OBI-2660, an aziridine bis-alkylating agent that causes crosslinking of DNA at the N7 (or O6) position of guanine, and subsequent tumor cell death [1]. The cytotoxicity of OBI-3424 is highly AKR1C3-dependent and this selective mode of activation distinguishes OBI-3424 from traditional prodrug alkylating agents, such as cyclophosphamide and ifosfamide, which are activated in the liver in a nonselective fashion.

In a study using 11 human hepatocellular carcinoma cell lines and 10 human NSCLC cell lines, OBI-3424 exhibited enhanced cytotoxicity in the cell lines with AKR1C3 overexpression compared with the cell lines with low (protein expression ≤0.21 and RNA expression ≤0.19 Log_2_FPKM [Crown Bio database]) or undetectable AKR1C3 expression [1]. OBI-3424 also showed antitumor activity as a single agent across various cell line-derived xenograft (HepG2, H460, VCap, SNU-16, and A498) and patient-derived xenograft (PA1280, GA6201, LU2505, and LU2057) models overexpressing AKR1C3. The IC_50_ values of OBI-3424 in cell lines expressing high AKR1C3 were in the low nanomolar range, indicating the high potency that is characteristic of a nitrogen mustard. Cytotoxicity was enhanced up to 5000-fold in the NCI-H2228 NSCLC cell line expressing high AKR1C3. This finding supports the strategy of targeting tumors with high expression of AKR1C3 while sparing low-AKR1C3-expressing regions found in normal tissues [1]. Thus, OBI-3424 has the potential to selectively treat AKR1C3-expressing tumors while sparing normal tissue.

We report the results of a Phase 1, first-in-human, sequential dose-escalation study of OBI-3424 in patients with advanced solid tumors. We evaluated the safety, tolerability, pharmacokinetics, pharmacodynamics, and preliminary efficacy of OBI-3424 as a single agent.

## Methods

The study was conducted at two centers in the United States: The University of Texas MD Anderson Cancer Center and The Ohio State University Comprehensive Cancer Center. All patients provided written informed consent at the time of enrollment stating that they were aware of the investigational nature of the study. The study was approved by the institutional review boards of both institutions and conducted in accordance with the ethical principles of the International Council for Harmonization Guideline for Good Clinical Practice, the Declaration of Helsinki, and applicable local regulations. The study was registered at www.clinicaltrials.gov (NCT03592264; July 19, 2018).

### Patients

Eligible patients were ≥18 years of age and had histologically or cytologically confirmed advanced solid tumors for which standard treatments were exhausted or were no longer effective; measurable disease as per Response Evaluation Criteria in Solid Tumors version 1.1 (RECIST v1.1) guidelines; Eastern Cooperative Oncology Group performance status of 0–1; and adequate hematologic, hepatic, and renal function. Patients were excluded if they had received radiotherapy to >25% of the bone marrow or had symptomatic brain metastases; other active malignancies (except for adequately treated nonmelanoma skin cancer, in situ cancer, or other cancers whose natural history or treatment did not have the potential to interfere with the safety or efficacy assessment of the study); active infection; radiotherapy, surgery, chemotherapy, targeted therapy, hormonal therapy, or an investigational drug/device within 28 days of the study start date; or concomitant use of strong CYP3A4 inhibitors/inducers or naproxen. A complete list of the inclusion and exclusion criteria is provided in the Supplementary Materials.

### Study design

A standard “3 + 3” dose-escalation design was used. The primary endpoints were incidence of dose-limiting toxicities (DLTs) and treatment-emergent adverse events (TEAEs), determination of the maximum tolerated dose (MTD)/recommended Phase 2 dose (RP2D), and characterization of the OBI-3424 pharmacokinetic profile. Secondary endpoints were tumor response as assessed by RECIST v1.1, and AKR1C3 expression in available tumor samples.

### Treatment

OBI-3424 was administered by intravenous infusion over 30 min. The starting dose of OBI-3424, 1 mg/m^2^, was selected on the basis of results of Good Laboratory Practice toxicology studies and pharmacologic and pharmacokinetic data obtained in the study of cynomolgus monkeys. This starting dose was administered on days 1 and 8 of each 21-day cycle, followed by escalation cohorts of 2, 4, 6, 8, and 12 mg/m^2^ (Schedule A). Following the observation of hematologic DLTs at the 12 mg/m^2^ dose level, the protocol was amended (Schedule B): escalation cohorts of 8, 10, 12, and 14 mg/m^2^ on day 1 of each 21-day cycle (i.e., once every 3 weeks). The MTD was defined as the dose level at which <2 of 6 patients experienced a DLT. Treatment was discontinued if there was clinically significant deterioration in the patient’s condition, disease progression, noncompliance/protocol violation, pregnancy, unacceptable toxicity, or consent withdrawal.

### Patient monitoring

Safety assessments were performed at least once every 3 weeks throughout the study treatment period and included physical examination, measurement of vital signs, clinical laboratory tests, 12-lead electrocardiography, and urinalysis. Cell counts, concomitant medication review, and collection of TEAEs were done weekly. TEAEs were graded according to the National Cancer Institute Common Terminology Criteria for Adverse Events, version 5.0.

Radiologic assessments of tumor response by computed tomography scan were conducted at baseline, every two cycles for the first six cyles, and at the end of every four cycles thereafter. Tumor response was measured using RECIST v1.1 [9].

A DLT was defined as the occurrence of any of the following events, within the first cycle of treatment, that was considered to be at least possibly related to OBI-3424: grade 4 neutropenia lasting ≥7 days; febrile neutropenia; grade 4 thrombocytopenia; grade 3 thrombocytopenia with ≥grade 2 bleeding requiring platelet transfusions; any other grade 3 or 4 nonhematologic toxicity (except grade 3 fatigue, nausea, vomiting, or diarrhea that resolved to grade 1 or baseline level within 72 h; grade 3 fever [in the absence of neutropenia]; grade 3 asymptomatic laboratory abnormalities that resolved to grade 1 or baseline level within 72 h or were not clinically significant; and asymptomatic grade 3 elevations in liver enzymes that resolved to grade 1 or baseline level within 7 days); and persistent aspartate aminotransferase/alanine aminotransferase levels of >3× the upper limit of normal with concomitant bilirubin value of >2× the upper limit of normal that was not clearly related to something other than the study drug. The period for DLT observation was 21 days from the first dose of OBI-3424.

### Pharmacokinetics

OBI-3424 (prodrug) and OBI-2660 (active metabolite) concentrations were analyzed from blood samples collected on day 1 of cycle 1 before, 15 min after the beginning of, and at the end of the infusion and 15, 30, 60, and 90 min and 2, 4, 6, and 8 h after the end of the infusion. Human plasma samples were analyzed by a validated method using liquid chromatography with tandem mass spectrometric detection. Plasma pharmacokinetic parameters were estimated with noncompartmental analysis using Phoenix WinNonlin Software (v8.3).

### Pharmacodynamics and biomarkers

**Fig. 2:**
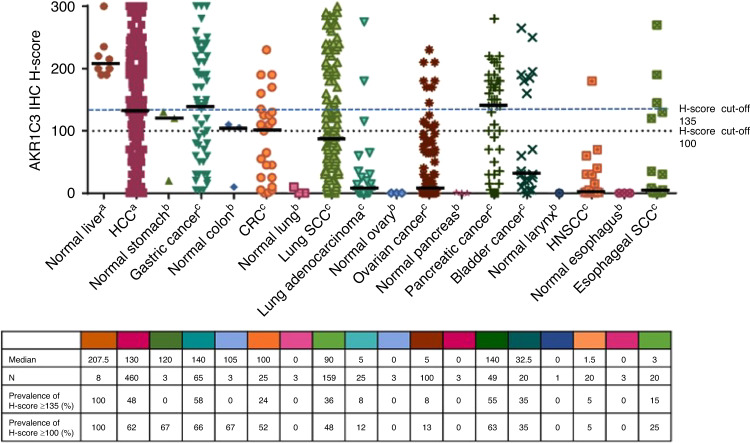
AKR1C3 expression level in 10 tumor tissue types. AKR1C3 aldo-keto reductase family 1 member C3, CRC colorectal carcinoma, HCC hepatocellular carcinoma, HNSCC head and neck squamous cell carcinoma, IHC immunohistochemistry, SCC squamous cell carcinoma. ^a^AKR1C3 IHC staining was performed on whole sections and tissue microarrays. ^b^AKR1C3 IHC staining was performed on tissue microarrays. ^c^AKR1C3 IHC staining was performed on whole sections. Black bar indicates median H-score; dotted lines indicate H-score cutoffs of 100 and 135.

Optional tumor tissue samples were collected prior to screening to measure AKR1C3 expression. OBI worked with the in vitro diagnostics company NeoGenomics Laboratories, Inc. (Aliso Viejo, CA, USA) to develop a validated immunohistochemistry (IHC) assay, which is being used to identify patients whose tumors have “high AKR1C3 expression” for inclusion in clinical trials with OBI-3424. Data from tissue microarrays or whole sections are summarized in Fig. 2. Normal liver, gastric, and colon tissues expressed moderate to high levels of AKR1C3, but expression of AKR1C3 in normal lung, ovary, pancreas, and esophagus tissues was minimal. Solid tumors across various sites of origin overexpressed AKR1C3, with hepatocellular carcinoma, gastric and pancreatic cancers, and squamous cell carcinomas of the lung and esophagus having the highest expression in the samples tested. Based on these data and the very tight positive association observed between cellular AKR1C3 expression and the IC_50_ of OBI-3424 observed in vitro, the initial AKR1C3 H-score cutoff for eligibility in the current study was amended from ≥135 to ≥100 to ensure recruitment of a patient population with tumors that had moderate to high AKR1C3 expression and therefore were theoretically more likely to respond to OBI-3424 therapy.

Tissue slides were considered evaluable if hematoxylin and eosin staining showed ≥100 viable tumor cells. AKR1C3 expression levels on consecutive tumor tissue slides were analyzed using the validated automated IHC assay on the Leica BOND-III platform (Leica Biosystems, Deer Park, Illinois, USA). Slides were incubated with anti-AKR1C3 antibody (Sigma-Millipore mouse monoclonal antibody, clone NP6.G6.A6, 1.2 µg/mL, 1:2000 dilution in BOND primary antibody diluent) or isotype control (Leica BOND ready-to-use negative control [mouse]) for 30 min. The Sigma-Millipore antibody has previously been validated against the following cell line controls: HCT 116 (negative control), genetically modified HCT 116 transfected with a derivative of plasmid F527-V5 EFα promoter to overexpress AKRIC3, Nalm (low endogenous expression), and TF1 (high endogenous expression) [9].

Reagents from the BOND Polymer Refine DAB Kit were used to perform the following steps on the Leica BOND-III IHC platform. Tumor AKR1C3 expression was evaluated using the H-score system [10], in which the percentages of AKR1C3-positive stained cells were recorded at each intensity (0, 1 + , 2 + , and 3 + ): intensity 0 implies no detectible staining, 1+ implies translucent or low-level expression, 2+ implies moderate or opaque staining, and 3+ implies strong or solid staining. The H-score values, ranging from 0 to 300, were calculated with the following formula: H-score = [(% of positive cells at intensity of 1 + ) × 1 + (% of positive cells at intensity of 2 + ) × 2 + (% of positive cells at intensity of 3 + ) × 3].

### Statistical analysis

The sample size in the current study was determined empirically, and there was no formal hypothesis testing. Categorical and continuous data were summarized with frequencies and percentages or descriptive statistics, respectively. All patients who received ≥1 dose of OBI-3424 (*n* = 39) were included in the safety analyses; the efficacy population included all patients in the safety population with a baseline assessment and ≥1 postbaseline tumor assessment.

## Results

### Patient demographics

From July 2, 2018, to July 20, 2021, 39 patients were enrolled and received ≥1 dose of OBI-3424. Patient demographics and baseline characteristics are displayed in Table 1. The median age was 67 years (range, 39–78 years). Patients were predominantly male (22/39, 56%), Caucasian/White (29/39, 74%), non-Hispanic/Latino (36/39, 92%), and had Eastern Cooperative Oncology Group performance status 1 (32/39, 82%). The most common tumor types were prostate (8/39, 21%) and colorectal (5/39, 13%) carcinoma.

**Table 1:** Demographics and baseline characteristics.

Variable	Day 1 and day 8 dosing					Day 1 dosing					
	Cohort 1 1.0 mg/m^2^ (*N* = 3)	Cohort 2 2.0 mg/m^2^ (*N* = 3)	Cohort 3 4.0 mg/m^2^ (*N* = 3)	Cohort 4 6.0 mg/m^2^ (*N* = 3)	Cohort 5 8.0 mg/m^2^ (*N* = 3)	Cohort 6 12.0 mg/m^2^ (*N* = 6)	Cohort 7 8.0 mg/m^2^ (*N* = 3)	Cohort 8 10.0 mg/m^2^ (*N* = 3)	Cohort 9 12.0 mg/m^2^ (*N* = 6)	Cohort 10 14.0 mg/m^2^ (*N* = 6)	Total (*N* = 39)
Females, *n* (%)	1 (33.3)	1 (33.3)	3 (100.0)	1 (33.3)	0	1 (16.7)	1 (33.3)	3 (100.0)	3 (50.0)	3 (50.0)	17 (43.6)
Age											3
Mean, year (SD)	71.3 (2.08)	64.3 (9.07)	58.3 (17.21)	53 (5.57)	65 (8.89)	68.3 (7.99)	54.7 (13.65)	60.7 (11.02)	59.8 (11.36)	68.2 (6.62)	63.1 (10.25)
Median, year	72	68	64	54	68	67.5	57	66	60	68.5	67
Min, Max, year	69, 73	54, 71	39, 72	47, 58	55, 72	55, 78	40, 67	48, 68	45, 75	59, 76	39, 78
Race											
African American, Black	0	0	0	0	1 (33.3)	1 (16.7)	0	0	0	2 (33.0)	4 (10.3)
Caucasian, White	2 (66.7)	2 (66.7)	3 (100)	3 (100)	1 (33.3)	5 (83.3)	3 (100)	3 (100)	4 (66.7)	3 (100)	29 (74.4)
Other	1 (33.3)	1 (33.3)	0	0	1 (33.3)	0	0	0	2 (33.3)	1 (16.7)	6 (15.4)
Ethnicity											
Hispanic, Latino	0	1 (33.3)	0	0	0	0	0	0	2 (33.3)	0	3 (7.7)
Non-Hispanic, Non-Latino	3 (100)	2 (66.7)	3 (100)	3 (100)	3 (100)	6 (100)	3 (100)	3 (100)	4 (66.7)	6 (100)	36 (92.3)
ECOG, *n* (%)											
0	0	0	1 (33.3)	0	0	1 (16.7)	2 (66.7)	0	1 (16.7)	2 (33.3)	7 (17.9)
1	3 (100.0)	3 (100.0)	2 (66.7)	3 (100.0)	3 (100.0)	5 (83.3)	1 (33.3)	3 (100.0)	5 (83.3)	4 (66.7)	32 (82.0)
Tumor type, *n* (%)											
Breast	0	0	0	0	0	0	0	0	0	1 (16.7)	1 (2.6)
Colorectal	0	0	0	1 (33.3)	0	0	0	0	1 (16.7)	3 (50.0)	5 (12.8)
Hepatocellular carcinoma	2 (66.7)	0	0	0	0	0	0	0	1 (16.7)	0	3 (7.7)
Lung	0	0	1 (33.3)	0	0	0	0	0	0	0	1 (2.6)
Melanoma	0	0	0	0	0	0	0	0	1 (16.7)	0	1 (2.6)
Ovarian	0	1 (33.3)	0	0	0	0	0	0	0	0	1 (2.6)
Prostate	0	2 (66.7)	0	0	1 (33.3)	3 (50.0)	2 (66.7)	0	0	0	8 (20.5)
Squamous cell carcinoma	0	0	0	0	2 (66.7)	0	0	0	0	0	2 (5.1)
Other	1 (33.3)	0	2 (66.7)	2 (66.7)	0	3 (50.0)	1 (33.3)	3 (100.0)	3 (50.0)	2 (33.3)	17 (43.6)
Stage, *n* (%)											
Stage 3	0	1 (33.3)	0	0	0	0	0	0	1 (16.7)	0	2 ()
Stage 4	3 (100.0)	2 (66.7)	3 (100.0)	3 (100.0)	3 (100.0)	6 (100.0)	3 (100.0)	3 (100.0)	5 (83.3)	6 (100.0)	37 (94.9)

*ECOG* Eastern Cooperative Oncology Group, *SD* standard deviation.

Patients were heavily pretreated. The median number of prior therapies was 5.7 (range, 2–15). Twenty-two patients (22/39, 56.4%) had prior surgery and 7 patients (7/39, 18.0%) had received prior radiation therapy.

Prior therapy included investigational drugs (*n* = 13 patients, 33.3%), either as monotherapy or in a combination regimen. Overall, 94.9% (37/39) of patients had received combination therapy (>50 combination regimens) and only 2 patients had received only monotherapy. Antimetabolites were the most widely administered class of drug with 21 patients receiving any of the following four drugs: 5-FU, capecitabine, gemcitabine, and pemetrexed. Six patients had received FOLFIRI, 8 had received FOLFOX, and 5 had received FOLFIRINOX.

Prior therapy included platinum agents in 69% (27/39) of patients or taxanes (paclitaxel, docetaxel, cabazitaxel, or nab-paclitaxel) in 54% (21/39) of patients. Of the 8 patients with prostate cancer, 6 had prior surgery, 8 had chemotherapy, and 7 patients were receiving androgen deprivation/hormonal therapy.

### Dose-limiting toxicity and maximum tolerated dose

In Schedule A, OBI-3424 was well tolerated at doses of up to 8 mg/m^2^. Hematologic DLTs (thrombocytopenia and anemia) were observed in all 6 patients who received a dose of 12 mg/m^2^ on Schedule A. These DLTs led to dose modifications and/or delays in subsequent cycles, and therefore, 8 mg/m^2^ was considered the MTD of OBI-3424 when administered as per Schedule A. Given that the platelet count nadirs were occurring on day 15 and day 21, the schedule of administration was modified to Schedule B. OBI-3424 administered every 3 weeks was tolerated at doses of up to 14 mg/m^2^ and the MTD was not reached. Overall, 19 patients required a red blood cell transfusion for grade ≥3 anemia, including 11 patients who required 2 or more separate red blood cell transfusions. Three patients receiving 14 mg/m^2^ OBI-3424 required a transfusion; 1 of these patients required 2 transfusions and 1 patient required 3 transfusions. Considering the significant burden associated with these transfusions, the RP2D and regimen of OBI-3424 were determined to be 12 mg/m^2^ every 3 weeks (Schedule B).

### Safety and tolerability

The median number of doses administered was 4 (range, 1–38). TEAEs occurred in 32 of the 39 patients (82%; Table 2); the most common TEAEs were anemia (25/39, 64%), thrombocytopenia (19/39, 49%), nausea (10/39, 26%), and fatigue (8/39, 21%). Gastrointestinal TEAEs were minimal. Nausea occurred in 10 patients (1 patient in each of the 1, 4, and 6 mg/m^2^ [Schedule A] cohorts, 1 patient in the 8 and 2 patients in each of the 10, 12, and 14 mg/m^2^ [Schedule B] cohorts), and only 5 patients reported vomiting (1 in the 4 mg/m^2^ [Schedule A] cohort, 3 in the 12 mg/m^2^ [Schedule A] cohort, and 1 in the 14 mg/m^2^ [Schedule B] cohort). Diarrhea occurred only at the higher doses of 12 mg/m^2^ (2 patients in each of the Schedule A and Schedule B cohorts) and 14 mg/m^2^ (Schedule B; 1 patient). Patients received prophylaxis with antinausea medications 1 h prior to the initial dose of OBI-3424; these medications included ondansetron at 8 mg intravenously and then orally every 8 h as needed or another 5-HT3 antagonist. Antidiarrheal therapies including dicyclomine (if predominant issue was cramping or abdominal pain), diphenoxylate/atropine and/or loperamide, and, if necessary, budesonide, were implemented on an as-needed basis.

**Table 2:** Patient incidence* of treatment-emergent adverse events (TEAEs).

TEAE, *n* (%)	Day 1 and day 8 dosing						Day 1 dosing				Total (*N* = 39)
	Cohort 1 1.0 mg/m^2^ (*N* = 3)	Cohort 2 2.0 mg/m^2^ (*N* = 3)	Cohort 3 4.0 mg/m^2^ (*N* = 3)	Cohort 4 6.0 mg/m^2^ (*N* = 3)	Cohort 5 8.0 mg/m^2^ (*N* = 3)	Cohort 6 12.0 mg/m^2^ (*N* = 6)	Cohort 7 8.0 mg/m^2^ (*N* = 3)	Cohort 8 10.0 mg/m^2^ (*N* = 3)	Cohort 9 12.0 mg/m^2^ (*N* = 6)	Cohort 10 14.0 mg/m^2^ (*N* = 6)	
Patients reporting any TEAE	3 (100.0)	1 (33.3)	3 (100.0)	2 (66.7)	3 (100.0)	6 (100.0)	3 (100.0)	3 (100.0)	6 (100.0)	6 (100.0)	36 (92.3)
Patients reporting any grade ≥3 TEAE	0	0	1 (33.3)	1 (33.3)	1 (33.3)	6 (100.0)	2 (66.7)	2 (66.7)	2 (33.3)	4 (66.7)	19 (48.7)
TEAEs in > 10% of patients											
Anemia	0	0	0	0	0	0	0	0	0	2 (33.3)	25 (64.1)
Thrombocytopenia	0	0	0	0	2 (66.7)	6 (100.0)	1 (33.3)	1 (33.3)	4 (66.7)	5 (83.3)	19 (48.7)
Lymphocytopenia	0	0	0	0	2 (66.7)	6 (100.0)	1 (33.3)	1 (33.3)	0	2 (33.3)	7 (17.9)
Leukopenia	0	0	0	1 (33.3)	0	2 (33.3)	0	0	3 (50.0)	3 (50.0)	7 (17.9)
Neutropenia	0	0	1(33.3)	0	0	1 (16.7)	0	0	2 (33.3)	2 (33.3)	6 (15.4)
Nausea	1 (33.3)	0	1 (33.3)	1 (33.3)	0	0	1 (33.3)	2 (66.7)	2 (33.3)	2 (33.3)	10 (25.6)
Diarrhea	0	0	0	0	0	2 (33.3)	0	0	2 (33.3)	1 (16.7)	5 (12.8)
Vomiting	0	0	1 (33.3)	0	0	3 (50.0)	0	0	0	1 (16.7)	5 (12.8)
Fatigue	1 (33.3)	0	0	1 (33.3)	1 (33.3)	1 (16.7)	0	2 (66.7)	2 (33.3)	0	8 (20.5)
Decreased appetite	0	0	0	0	0	3 (50.0)	0	0	0	1 (16.7)	4 (10.3)
Dyspnea	0	0	0	0	0	1 (16.7)	0	1 (33.3)	0	2 (33.3)	4 (10.3)

^*^Patients reporting multiple TEAEs with the same System Organ Class or Preferred Term are counted only once in that row.

Four patients experienced dyspnea at higher doses of OBI-3424: 1 patient each in the 10 mg/m^2^ (Schedule B) and 12 mg/m^2^ (Schedule A) cohorts and 2 patients in the 14 mg/m^2^ (Schedule B) cohort. Dyspnea was determined not to be related to treatment with OBI-3424. A 78-year-old male patient with carcinoma of the prostate required 2 separate red blood cell transfusions for worsening anemia and low hemoglobin, which were likely the cause of the dyspnea in this patient. A 66-year-old female patient had anemia, pleural effusion, and pneumothorax (one-third of right lower lobe with diminished sounds and faint crackling), all of which may have contributed to dyspnea in this patient. The dyspnea in a 66-year-old male patient and a 63-year-old female patient, both with colorectal cancer, was likely due to worsening anemia, and in the case of the female patient, a mitral valve prolapse and hypoalbuminemia.

Nineteen patients (49%) had grade ≥3 TEAEs, including anemia (16/39, 41%), thrombocytopenia (10/39, 26%), neutropenia (3/39, 8%), and lymphocytopenia (2/39, 5%). Three patients, all of whom received OBI-3424 at 12 mg/m^2^ on Schedule A, experienced grade ≥3 anemia as a serious TEAE, and 2 of these patients also experienced grade ≥3 thrombocytopenia as a serious TEAE. Nineteen patients required transfusions; 10 of those 19 patients were receiving doses ≥12 mg/m^2^ (5/6 patients receiving 12 mg/m^2^ on Schedule A, 2/6 patients receiving 12 mg/m^2^ on Schedule B, and 3/6 patients receiving 14 mg/m^2^ on Schedule B).

Eleven patients (28%) required dose interruption and/or reduction due to TEAEs. Ten patients (26%) experienced dose reductions due to thrombocytopenia, anemia, neutropenia, and fatigue, and 4 patients (10%) experienced dose interruptions due to anemia, thrombocytopenia, dehydration, and orthostatic hypotension. Two patients (5%) discontinued treatment. One patient, a 54-year-old male patient with castrate-resistant prostate cancer metastatic to the bone without visceral metastases, who received 3 doses of OBI-3424 at 2 mg/m^2^ on Schedule A, experienced a grade 3 right cerebral subdural hematoma. The patient had received treatment for 27 days when he presented to the emergency department following several falls that resulted in confusion. On admission, a computed tomography (CT) scan revealed a right cerebral subdural hematoma, and the patient underwent an emergency craniotomy. The next day, the patient was doing relatively well with no neurologic deficits and no headache. Four days later, a CT scan revealed an increase in the hematoma, and another craniotomy was performed. The day after surgery, the patient developed a right frontal hematoma with subsequent inability to move the upper left extremity, facial droop, dysarthria, and seizures. Two days later, a CT scan showed increased hemorrhaging and edema, which the hematologist considered to be related to steroid therapy for cytopenia. The patient was discharged to hospice care in stable condition 1 day later. Ten days after discharge, the patient suffered another subdural hematoma and died. During hospitalization, the patient received 2 separate transfusions 14 days apart due to worsening anemia and thrombocytopenia. The subdural hematomas were determined to be due to head trauma resulting from several falls and were not related to treatment with OBI-3424.

The second patient discontinued treatment with OBI-3424 due to leukemia secondary to oncologic chemotherapy (the patient had received extensive previous treatments, including DNA-alkylating agents, and had received only 1 dose of OBI-3424 at the 4.0 mg/m^2^ dose level; therefore this adverse event was also not attributed to the treatment drug). There were no grade 5 drug-related TEAEs.

**Fig. 3:**
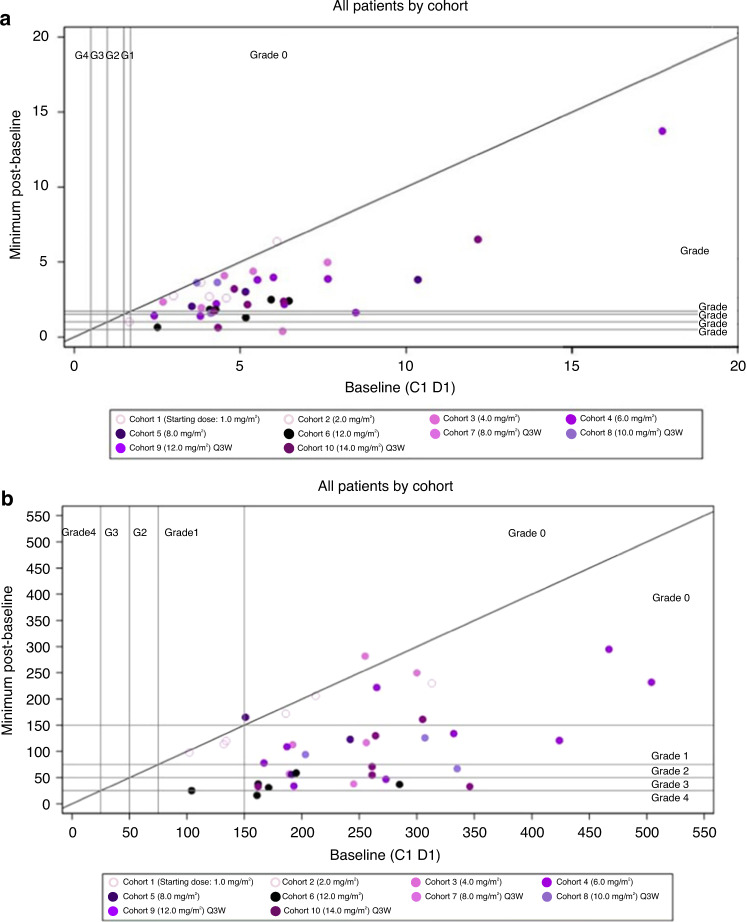
Hemoglobin level and platelet count changes with treatment. Change in (**a**) hemoglobin value and (**b**) platelet count from baseline to the minimum observed postbaseline value. Solid diagonal line represents no change; vertical and horizontal lines are borders between NCI CTCAE grades. Vertical distance from the diagonal represents magnitude of change; above the diagonal is an increase, below a decrease. For analytes in which the reference range varied by age/sex, the lowest value was used to create the borders. Color gradient indicates dose intensity (lighter = lower dose intensity, darker = higher dose intensity). C1 D1 cycle 1, day 1, NCI CTCAE National Cancer Institute Common Terminology Criteria for Adverse Events, Q3W every 3 weeks.

Treatment with OBI-3424 at doses of ≥8 mg/m^2^ using Schedule A and ≥12 mg/m^2^ using Schedule B was associated with grade ≥3 anemia and thrombocytopenia. Changes in hemoglobin and platelet counts during treatment are illustrated in Fig. 3a, b.

### Pharmacokinetics

**Fig. 4:**
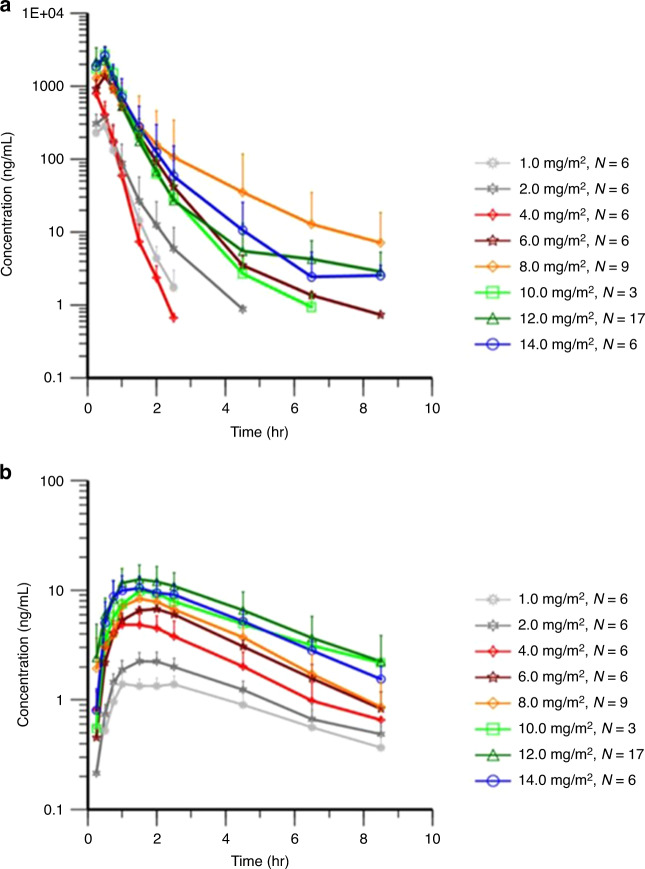
Pharmacokinetic profiles. **a** OBI-3424 and **b** OBI-2660 during cycle 1 (days 1 and 8 combined).

Mean plasma concentration versus time profiles of single doses of OBI-3424 and its active metabolite (OBI-2660) at doses ranging from 1 to 14 mg/m^2^ are illustrated in Fig. 4a, b. Pharmacokinetic profiles revealed that OBI-3424 clearance varied widely between patients and across dose levels, whereas the concentration of OBI-2660 increased with increasing dose and exhibited less variation across dose levels.

OBI-3424 and OBI-2660 pharmacokinetic parameters by dose level are summarized in Supplementary Table S1 (Supplementary Material). Maximum serum concentrations (*C*_max_) of OBI-3424 generally occurred shortly after the 30-min drug infusion (between 33 and 50 min). The time to maximum concentration of the active metabolite OBI-2660 was observed to be slightly delayed compared with that for OBI-3424, with the *C*_max_ achieved between 1.33 h and 1.75 h after the start of drug infusion. Exposures (*C*_max_ and area under the concentration-time curve [AUC]) to OBI-3424 increased with increasing doses; however, an analysis of dose proportionality for OBI-3424 using the power model was inconclusive. Exposures to OBI-2660 increased across the dose range of 1 mg/m^2^ to 14 mg/m^2^ but in a less than proportional manner.

Exposure (AUC) of OBI-2660 was much lower than that of OBI-3424, with the AUC approximately 1.4% to 3.4% of OBI-3424. The half-life of OBI-3424 was short (0.21–0.74 h), whereas OBI-2660 had a longer half-life (1.87–3.08 h). Mean clearance of OBI-3424 ranged from 4.8 L/h/m^2^ to 8.9 L/h/m^2^, and volume of distribution at steady state ranged from 2.5 L/m^2^ to 4.3 L/m^2^ for OBI-3424. No accumulation of exposure (*C*_max_ and AUC) between 2 doses (cycle 1 day 1 and cycle 1 day 8) was observed for either OBI-3424 or OBI-2660.

### Pharmacodynamics

**Fig. 5:**
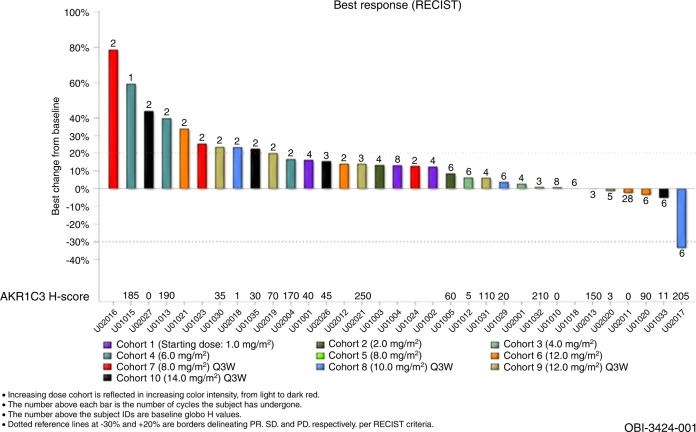
Antitumor activity in patients treated with OBI-3424 at postbaseline scans.

Thirty-two of the 39 patients provided archival (28/32) or fresh biopsy (4/32) tumor tissue specimens for AKR1C3 expression testing using the validated, automated IHC assay (NeoGenomics). Among the 32 specimens, 6 tumor tissue specimens did not have sufficient viable tumor cells and were non-evaluable. AKR1C3 H-scores by patient are listed in Fig. 5.

### Antitumor activity

Thirty-three of 39 patients were evaluable for tumor response by RECIST v1.1. Six patients were not evaluable because of early discontinuation due to disease progression (*n* = 2), consent withdrawal (*n* = 2), clinically significant deterioration in the patient’s condition (*n* = 1), or death (*n* = 1).

A patient with cholangiocarcinoma, treated with OBI-3424 at 10 mg/m^2^ (Schedule B), had a partial response (33% reduction in tumor measurements). Stable disease lasting for ≥6 weeks was noted in 21 patients (64%), with 15/21 patients (71%) experiencing stable disease for ≥4 cycles (12 weeks), including a patient with esthesioneuroblastoma who remained on study for >28 cycles (12 mg/m^2^ OBI-3424, Schedule A, dose reduced to 9 mg/m^2^ every 3 weeks after 4 cycles). The remaining 11 patients (33%) had progressive disease. Best response by RECIST v1.1 is shown in Fig. 5.

Reasons for discontinuation of the study drug for all patients were as follows: disease progression (*n* = 27), consent withdrawn (*n* = 5), clinically significant deterioration in the patient’s condition (*n* = 4), death (subdural hematoma unrelated to study drug; *n* = 1), logistical reasons (unable to travel to receive treatment due to COVID-19 protocols; *n* = 1), and leukemia secondary to chemotherapy (unrelated to study drug; *n* = 1).

## Discussion

In this first-in-human, dose-escalation, Phase 1 study, OBI-3424, a small-molecule prodrug, was tolerated at doses of up to 14 mg/m^2^ when administered as per Schedule B. No DLTs occurred at the maximum administered dose, and thus, the MTD was not determined. All patients treated with OBI-3424 at 12 mg/m^2^ on Schedule A experienced anemia and/or thrombocytopenia that required blood transfusions and dose reductions. These TEAEs, especially at doses of ≥8 mg/m^2^ (Schedule A) or ≥12 mg/m^2^ (Schedule B), led to the determination that the RP2D should be 12 mg/m^2^ every 21 days (Schedule B), at which dose OBI-3424 was generally well tolerated, with manageable toxicities.

The most common TEAEs were anemia (64%), thrombocytopenia (49%), nausea (26%), and fatigue (21%). Three patients experienced serious TEAEs; all 3 experienced grade ≥3 anemia and 2 of the 3 also experienced grade ≥3 thrombocyopenia. The occurrence of transient thrombocytopenia is consistent with published data on AKR1C3 overexpression in normal bone marrow. In a recent study, 20 normal archival bone marrow samples from patients with B-cell acute lymphoblastic leukemia (ALL) and T-cell ALL were evaluated for AKR1C3 expression by IHC, protein Western blotting, and quantitative reverse-transcriptase PCR. An H-score was used to quantify the percentage of nuclear immunoreactivity for AKR1C3 with varying disease involvement. T-cell ALL samples had higher H-scores (172–190) than B-cell ALL samples (30–160). Follow-up IHC assessment of normal marrow specimens revealed that AKR1C3 is expressed in a subset of normal bone marrow cells that morphologically resemble erythroid lineage cells [11]. These observations may explain the risk of anemia and thrombocytopenia observed during the dose-escalation phase of the current study and demonstrate the on-target toxicity of OBI-3424.

OBI-3424 exhibited linear, dose-proportional pharmacokinetics at doses ranging from 1 mg/m^2^ to 14 mg/m^2^ without marked accumulation after repeated dosing. Circulating levels of the metabolite OBI-2660 were low relative to OBI-3424, accounting for 1.4% to 3.4% of OBI-3424 plasma exposure. OBI-3424 exposures at the RP2D exceeded the target exposure (454 h ng/mL, unpublished data), which exerts antitumor activity in human tumor xenograft models. A clear pharmacokinetic/pharmacodynamic relationship was observed between OBI-3424 AUC and maximum platelet reduction in cycle 1 across all cohorts, whereas the correlation between OBI-2660 serum exposure and decreased serum platelet count was insignificant. Notably, highly variable exposure of both OBI-3424 and OBI-2660 was observed across 6 patients given the RP2D dose of 12 mg/m^2^. This high inter-subject variation in OBI-3424 exposure (AUC ranged from 5,805 to 1,323, a 4.38-fold difference from highest to lowest) may have resulted in limited tumor response in the patients who had lower exposure. Further pharmacokinetic data from an ongoing Phase 2 dose-expansion study may help elucidate the clinical results as a function of the pharmacokinetic profiles of both OBI-3424 and OBI-2660.

The best response to treatment with OBI-3424 was a partial response. One patient with cholangiocarcinoma had a partial response with 33% tumor reduction by local assessment while receiving OBI-3424 at a dose of 10 mg/m^2^ as per Schedule B. Of note, this patient had particularly high AKR1C3 expression, with an IHC H-score of 205. Although the mechanism of action of OBI-3424 is AKRIC3-dependent, AKR1C3 expression was not an inclusion criterion for this dose-escalation study, and as a result, most study patients had minimal AKR1C3 expression (mean H-score was 82), which may have contributed to the limited antitumor activity observed. Eight of the patients had prostate cancer and their median age of 67 years may have made them more vulnerable to toxicity, resulting in more frequent dose reductions, which may have also contributed to the limited antitumor activity observed in the current study.

AKR1C3 is overexpressed in 90% of hepatocellular tumors and in 50% of pancreatic tumors, and there is a significant unmet need for better treatments in these cancers, especially for patients whose disease has progressed after surgery and/or systemic chemotherapy. Thus, a Phase 2 study of single-agent OBI-3424 is currently enrolling patients with locally advanced/metastatic pancreatic cancer, hepatocellular cancer, or other epithelial carcinomas with tumoral AKR1C3 overexpression.

## Supplementary information

Tsimberidou et al Supplementary Materials

## Data Availability

The data generated in the current study are available within the article and its supplementary data files.
