# Supplementary material for: Phase 1 dose-escalation study evaluating the safety, pharmacokinetics, and clinical activity of OBI-3424 in patients with advanced or metastatic solid tumors
Source: Br J Cancer. 2023 May 12;129(2):266–74. doi: 10.1038/s41416-023-02280-4 (PMC10180615; doi:10.1038/s41416-023-02280-4)
Supplement: Supplementary file 1 — Tsimberidou et al Supplementary Materials [file 41416_2023_2280_MOESM1_ESM.docx]

# Tsimberidou et al. Phase 1 Dose-Escalation Study Evaluating the Safety, Pharmacokinetics, and Clinical Activity of OBI-3424 in Patients with Advanced or Metastatic Solid Tumors

# Supplementary Materials

##

## Inclusion Criteria

1. At least 18 years of age
2. Ability to understand the purposes and risks of the study and has signed a written informed consent form approved by the investigator’s Institutional Review Board/Independent Ethics Committee
3. Recovered from toxicities of prior therapy to grade 0 or 1
4. Measurable disease by Response Evaluation Criteria in Solid Tumors version 1.1 criteria
5. Eastern Cooperative Oncology Group performance status of 0 or 1
6. Acceptable liver function:
   1. Bilirubin value of ≤1.5 × institutional upper limit of normal (ULN)
   2. Alanine aspartate levels and alanine aminotransferase levels of ≤3.0 × ULN, or ≤5.0 × ULN for subjects with liver involvement
7. Acceptable renal function:

a. Creatinine clearance of >30 mL/min according to the Cockcroft-Gault formula

1. Acceptable hematologic status (without hematologic support, other than red blood cell transfusion):
   1. Absolute neutrophil count of ≥1500 cells/μL
   2. Platelet count of ≥100,000/μL
   3. Hemoglobin level of ≥9.0 g/dL (prior packed red blood cell transfusion or erythropoietin support is allowed)
2. Females of childbearing potential must not have had unprotected sexual intercourse within 30 days before study entry and must agree to use a highly effective method of contraception (eg, total abstinence, an intrauterine device, a double-barrier method [such as condom plus diaphragm with spermicide], a contraceptive implant, an oral contraceptive, or have a vasectomized partner with confirmed azoospermia) throughout the entire study period and for 30 days after study drug discontinuation
3. Histologically or cytologically confirmed solid malignancy that is metastatic or unresectable and for which standard curative or palliative measures do not exist or are no longer effective
4. Tumor progression after most recent therapy

## Exclusion Criteria

1. Prior radiotherapy to >25% of the bone marrow
2. Symptomatic brain metastases, unless previously treated and well controlled for ≥4 weeks after central nervous system–directed treatment as ascertained by clinical examination and brain imaging (magnetic resonance imaging or computed tomography) during the Screening Period. Patients with known leptomeningeal disease are excluded
3. Previously treated malignancies, except for adequately treated nonmelanoma skin cancer, *in situ* cancer, or other cancers whose natural history or treatment does not have the potential to interfere with the safety or efficacy assessment of the current study
4. Patients with hepatocellular carcinoma (applies to Expansion Phase only)
5. Major surgery, other than diagnostic surgery, within 4 weeks prior to day 1, without complete recovery
6. Active, uncontrolled bacterial, viral, or fungal infections requiring systemic therapy
7. Treatment with radiation therapy, surgery, chemotherapy, targeted therapies, or hormonal therapy within 3 weeks or 5 half-lives, whichever is shorter, prior to study entry (6 weeks for nitrosoureas or mitomycin C)
8. Concomitant use of strong CYP3A4 inhibitors/inducers
9. Concomitant use of naproxen within a 48-hour window before and after OBI-3424 dosing
10. Females who are pregnant or breastfeeding
11. Concomitant disease or condition that could interfere with the conduct of the study, or that would, in the opinion of the investigator, pose an unacceptable risk to the subject in this study
12. Unwillingness or inability to comply with the study protocol for any reason

Table S1. Mean Plasma Pharmacokinetic Parameters for OBI-3424 and OBI-2660 in Cycle 1 ― Days 1 and 8 Combined

| **Dose level**  **(mg/m^2^)** | **T_max_**  **(h)** | | | | **C_max_**  **(ng/mL)** | | | | **AUC_0-t_**  **(h*ng/mL)** | | | | **T_1/2_**  **(h)** | | | | **CL**  **(L/h/m^2^)** | | **Vd_ss_**  **(L/m^2^)** | |
| --- | --- | --- | --- | --- | --- | --- | --- | --- | --- | --- | --- | --- | --- | --- | --- | --- | --- | --- | --- | --- |
|  | **OBI-3424** | | **OBI-2660** | | **OBI-3424** | | **OBI-2660** | | **OBI-3424** | | **OBI-2660** | | **OBI-3424** | | **OBI-2660** | | **OBI-3424** | | **OBI-3424** | |
| 1 | 0.46 | (0.10) | 1.75 | (0.61) | 289.30 | (56.10) | 1.70 | (0.80) | 217.10 | (46.10) | 7.30 | (1.90) | 0.29 | (0.04) | 3.08 | (1.21) | 4.74 | (0.87) | 2.45 | (0.40) |
| 2 | 0.50 | 0.00 | 1.67 | (0.26) | 375.80 | (161.40) | 2.30 | (0.50) | 298.00 | (156.50) | 10.00 | (2.20) | 0.31 | (0.14) | 2.48 | (0.76) | 8.85 | (5.25) | 4.13 | (1.67) |
| 4 | 0.33 | (0.13) | 1.33 | (0.41) | 803.50 | (500.70) | 5.00 | (1.10) | 650.90 | (391.60) | 19.30 | (6.70) | 0.21 | (0.04) | 2.00 | (0.24) | 7.74 | (3.29) | 2.68 | (1.69) |
| 6 | 0.50 | 0.00 | 1.75 | (0.27) | 1360.30 | (247.10) | 6.80 | (0.40) | 1278.10 | (230.00) | 27.40 | (3.50) | 0.74 | (0.39) | 2.09 | (0.44) | 4.84 | (1.03) | 3.72 | (0.83) |
| 8 | 0.42 | (0.13) | 1.44 | (0.30) | 1613.10 | (419.80) | 8.50 | (1.60) | 1678.40 | (1302.00) | 32.80 | (8.90) | 0.67 | (0.33) | 1.87 | (0.35) | 6.53 | (2.92) | 3.97 | (1.06) |
| 10 | 0.50 | 0.00 | 1.67 | (0.29) | 2654.70 | (426.40) | 10.10 | (1.10) | 2037.00 | (316.20) | 29.20 | (18.30) | 0.57 | (0.24) | 2.99 | (0.10) | 4.92 | (0.83) | 3.06 | (0.50) |
| 12 | 0.44 | (0.17) | 1.53 | (0.37) | 2481.90 | (1132.70) | 13.10 | (4.20) | 2159.40 | (1089.10) | 56.60 | (21.40) | 0.55 | (0.34) | 2.41 | (0.69) | 8.20 | (7.98) | 4.33 | (4.67) |
| 14 | 0.42 | (0.13) | 1.75 | (0.52) | 2619.20 | (878.00) | 11.20 | (2.70) | 2131.80 | (955.10) | 45.60 | (9.80) | 0.55 | (0.27) | 2.46 | (0.78) | 8.49 | (5.67) | 4.31 | (1.22) |

Data expressed as mean (standard deviation).
AUC_0-t_, area under the concentration-time curve from time 0 to last time point measured; CL, clearance; C_max_, maximum concentration; T_1/2_, half-life; T_max_, maximum time; Vd_ss_, volume of distribution at steady state.
